# Analysis of TNFAIP3, IRAK1, and TLR4 Gene Polymorphisms in Patients With Rheumatoid Arthritis

**DOI:** 10.1002/iid3.70344

**Published:** 2026-02-12

**Authors:** Zhenboyang Tang, Lihui Peng, Zixia Zhao, Xiping Zhou, Xiru Ling, Chunyan Huang, Jiqiang Wu, Ping Wang, Jie Chen

**Affiliations:** ^1^ Department of Rheumatology and Immunology The Affiliated Hospital of Southwest Medical University Luzhou P.R. China; ^2^ Department of Rheumatology and Immunology The Third Hospital of Mianyang (Sichuan Mental Health Center) Mianyang China; ^3^ Department of Rheumatology and Immunology The Second People's Hospital of Meishan City Meishan Sichuan China; ^4^ Stem Cell Immunity and Regeneration Key Laboratory of Luzhou P.R. China

**Keywords:** IRAK1, rheumatoid arthritis, single nucleotide polymorphism, TLR4, TNFAIP3

## Abstract

**Objective:**

Rheumatoid arthritis (RA) arises from a complex interplay of polygenic susceptibility, epigenetic factors, and environmental modifiers. This study investigated the correlation between RA and Tumor necrosis factor α‐induced protein 3 (TNFAIP3), Interleukin‐1 receptor‐associated kinase 1 (IRAK1), and Toll‐like receptor 4 (TLR4) gene polymorphisms.

**Methods:**

A sample of 308 RA cases and 310 matched controls was included. Genotyping of the TNFAIP3, IRAK1, and TLR4 was performed via mass spectrometry.

**Results:**

Compared to healthy controls, individuals carrying the rs1059703 A allele (odds ratio (OR) = 0.640, 95% confidence interval (CI): 0.458–0.895, *p* = 0.009) of IRAK1, individuals carrying the rs5029930 C allele (OR = 0.469, 95% CI: 0.298–0.739, *p* = 0.001), and AC genotype (OR = 0.427, 95% CI: 0.255–0.716, *p* = 0.001) of TNFAIP3 had a lower RA risk. Individuals carrying the rs5029939 C allele (OR = 2.401, 95% CI: 1.436–4.017, *p* = 0.001) of TNFAIP3, individuals carrying the rs7873784 C allele (OR = 1.549, 95% CI: 1.101–2.179, *p* = 0.012), and CG genotype (OR = 1.489, 95% CI: 1.011–2.193, *p* = 0.043) of TLR4 had a higher RA risk.

**Conclusion:**

In summary, the TNFAIP3, IRAK1, and TLR4 gene polymorphisms are associated with RA susceptibility.

**Abbreviations**
ACPA: anti‐cyclic citrullinated peptide antibody
ACR: American College of Rheumatology
CI: confidence interval
DAS28: disease activity score 28
GH: general health
GWAS: genome‐wide association study
IRAK1: interleukin‐1 receptor associated kinase 1
NF‐κB: nuclear factor κB
OR: odds ratio
RA: rheumatoid arthritis
RF: rheumatoid factor
SJC: swollen joint counts
SNPs: single nucleotide polymorphisms
TJC: tender joint counts
TLR4: toll‐like receptor 4
TNFAIP3: tumor necrosis factor α‐induced protein 3

## 1 Introduction

Rheumatoid arthritis (RA) is a disease that can cause damage to multiple systems. The defining characteristic of RA is synovitis, which is associated with immune disorders [1]. Although the exact etiological mechanism of RA is currently unknown, current research suggests that the interaction between environmental factors and genetic background can increase the risk of developing the disease [2]. Single nucleotide polymorphisms (SNPs) have been extensively studied to explain human genetic variations. In particular, SNPs related to diseases have gained significant attention in the field of genetics and have been widely utilized in the research of polygenic diseases [3]. With the advancements in genotyping and sequencing methods, numerous loci implicated in RA pathogenesis have been investigated. Notably, genome‐wide association studies (GWAS) have validated over 100 RA risk loci in Europe and Asia [4].

TNFAIP3 encodes a ubiquitin‐editing enzyme that terminates NF‐κB activation by deubiquitinating key signaling molecules [5]. NF‐κB and pro‐inflammatory factors, such as IL‐6 and TNF‐α, have the ability to mutually enhance each other, thereby aggravating the initiation and advancement of RA [6]. TNFAIP3‐deficient mice have been found to exhibit severe arthritis [7]. Furthermore, mice lacking TNFAIP3 in bone marrow cells develop destructive arthritis with characteristics similar to RA [8]. TNFAIP3 defects have been implicated in the occurrence of various autoimmune disorders in humans [9]. Studies have suggested that SNPs in the TNFAIP3 region are associated with RA [10]. Plenge et al.'s study, conducted on Americans, confirmed the genetic association between rs10499194 and rs6920220 in the TNFAIP3 region and RA [11]. Thomson et al.'s study provided evidence that gene polymorphisms in the TNFAIP3 region in the British population are linked to RA [12]. Benahmed D et al.'s study in the Algerian population suggested that TNFAIP3 is not associated with susceptibility to RA [13].

IRAK1 is a pivotal kinase downstream of TLR signaling, resulting in the upregulation of inflammatory cytokines and chemokines [14]. The importance of IRAK1 in TLR signaling and inflammation is further underscored by studies showing that IRAK1 deficiency or inhibition leads to a significant reduction in inflammatory responses. For instance, IRAK1‐deficient mice exhibit reduced cytokine production and inflammation in response to TLR ligands [15]. One of the earliest studies to investigate the association between IRAK1 gene polymorphisms and RA was conducted in an Egyptian population [16]. This study found a strong association between a SNP in the IRAK1 gene (rs3027898) and RA susceptibility. Several meta‐analysis studies have been conducted to pool data from multiple studies and increase statistical power. One meta‐analysis study revealed a noteworthy correlation between rs3027898 and RA susceptibility in a Caucasian population, but not in an Asian population [17]. Another meta‐analytical study reported a significant correlation between rs1059703 and RA susceptibility in an Asian population, but not in a Caucasian population [18]. These findings suggest potential variations in the genetic architecture of RA among different ethnic groups.

The importance of TLR4 in innate immunity and inflammation is underscored by studies showing that TLR4 deficiency leads to a significant reduction in inflammatory responses to LPS and other TLR4 ligands. A significant amount of research has been conducted on the correlation between TLR4 gene polymorphisms and the vulnerability to RA. These studies have reported associations between various SNPs in the TLR4 gene, including rs4986791 (the Thr399Ile polymorphism), rs4986790, and rs1927911, and RA susceptibility [19, 20]. However, the findings of these studies have displayed inconsistency. One of the initial studies examining the association between TLR4 gene polymorphisms and RA did not find any association between a SNP in the TLR4 gene (rs4986790) and RA susceptibility [21].

Based on the aforementioned research background and the genetic heterogeneity observed among different races, this study explores the associations between the gene loci of TNFAIP3 (rs6920220, rs5029930, and rs5029939), IRAK1 (rs1059703), and TLR4 (rs1927914 and rs7873784) and RA susceptibility in the Chinese Han population.

## 2 Materials and Methods

### 2.1 Participants

The study encompassed a sample of 618 participants of Han Chinese descent, with 308 RA cases from the Affiliated Hospital of Southwest Medical University and 310 healthy controls matched for age and gender. Participants were selected based on specific criteria, which included: (1) all individuals included in the study were of Chinese Han ethnicity; (2) all cases in the case group met the 2010 American College of Rheumatology (ACR) classification criteria for RA; and (3) there was no genetic association among any of the participants. The exclusion criteria included the following: (1) patients who had recently used non‐steroidal anti‐inflammatory drugs, glucocorticoids, immunosuppressants, biologics, or any other medications; (2) patients with other autoimmune diseases, mental illnesses, infectious diseases, malignant tumors, acquired immunodeficiency syndrome, or any other life‐threatening conditions. The basic information of all participants, including name, gender, age, nationality, and ethnicity, was collected and organized. Comprehensive clinical and experimental data were gathered from the RA, including rheumatoid factor (RF), anti‐cyclic citrullinated peptide antibodies (ACPA), disease activity score 28 (DAS 28), general health (GH) scores, tender joint count (TJC) for 28 joints, swollen joint count (SJC), and morning stiffness. Prior to enrollment, all study subjects provided their informed consent by signing a consent form, and a 5 mL blood sample was collected after enrollment.

This study has received approval from the Ethics Research Committee of Southwest Medical University.

### 2.2 DNA Extraction and Genotyping

DNA extraction was conducted in accordance with the instructions provided by the blood DNA extraction kit manufactured by Tiangen Company (Beijing, China). SNP genotyping was conducted using flight‐time mass spectrometry. Its key feature is as follows: After multiple PCR amplifications, the products are amplified by adding SNP sequence‐specific extension primers. At the SNP site, one base is extended, with different genotypes extending different bases. Subsequently, under intense nanosecond (10⁻⁹ s) laser pulses, particles are separated within a non‐drift electric field region based on their mass‐to‐charge ratio. The time required for particles carrying extended bases to travel through a vacuum tube to the detector varies according to their mass, enabling genotype differentiation. Primer sequences for PCR amplification and single base extension were designed using Sequenom's MassARRAY Assay Design software. Detailed information about the primer can be found in Supporting Information Table 1. The obtained genotyping data was analyzed using TYPER software to analyze the experimental results.

### 2.3 Statistical Analysis

Statistical analysis was conducted by SPSS 25 statistical software. Statistical significance was determined when the bilateral *p*‐value was less than 0.05. After conducting the test, it was found that the age of the research subjects included in this study followed a normal distribution. Therefore, the mean ± standard deviation (SD) was utilized to represent the data, and variance analysis was employed to compare the age differences between genotypes within the RA group. The gender of the research subjects was expressed in terms of frequency (*n*) and percentage (%), and the chi‐square (*χ*2) test was utilized to evaluate the differences in gender among different genotypes in the RA group. Binary logistic regression was employed to investigate the association between the polymorphisms at different loci of TNFAIP3, IRAK1, and TLR4 and RA susceptibility, laboratory data, and clinical symptoms. The correlations between the alleles and genotypes and RA susceptibility, laboratory data, and clinical symptoms were analyzed using the *χ*2 test or one‐way ANOVA. The OR and 95% CI were calculated.

The chi‐square test was utilized to evaluate whether the samples included in the study adhered to the Hardy–Weinberg equilibrium (HWE) test. The obtained *p*‐value was greater than 0.05, suggesting that the selected group was representative of the larger group.

## 3 Results

### 3.1 General Data Analysis of the Research Object

The findings indicated that the RA case group and control group were balanced regarding gender (*p* = 1.000) and age (*p* = 0.223). The results are presented in Table 1.

**TABLE 1:** General data of the RA case group and control group.

Common data	Healthy controls *n* = 310	RA *n* = 308	*p*
Gender (male/female)	83/227	82/226	1.0000
Age (years)	52.54 ± 11.99	53.64 ± 10.37	0.223
RF	—	212.86 ± 200.94	—
ACPA (positive/negative)	—	283/25	—
DAS28 (light/medium/heavy)	—	6/74/228	—
Tender joint (*n*)	—	10.32 ± 7.81	—
Swelling joints (*n*)	—	6.97 ± 7.17	—
GH	—	74.53 ± 14.18	—
Morning stiffness (with/without)	—	225/83	—

Abbreviations: ACPA, anti‐cyclocitrulline peptide antibody; DAS28, disease activity score 28; GH, general health score; n, number of participants; RF, rheumatoid factor.

### 3.2 Hardy–Weinberg Equilibrium (HWE) Test

The genetic polymorphisms of TNFAIP 3, IRAK 1, and TLR 4 all adhered to the HWE test (*p* > 0.05), suggesting that the chosen study participants were representative (Table 2).

**TABLE 2:** Distribution and HWE test of gene loci in the RA case group and control group.

		Healthy controls				RA group			
		Actual frequency	Theory frequency	*χ*²	*p*	Actual frequency	Theory frequency	*χ*²	*p*
TNFAIP3 rs6920220 G/G		305	303.22	3.607	0.165	304	302.11	4.673	0.097
A/G		3	6.74			1	4.87		
A/A		2	0.04			2	0.02		
rs5029930 A/A		283	280.72	1.477	0.478	252	249.94	0.662	0.718
A/C		24	28.55			50	54.13		
C/C		3	0.73			5	2.93		
rs5029939 C/C		290	288.38	2.236	0.327	264	260.01	2.792	0.248
C/G		18	21.23			38	45.96		
G/G		2	0.39			6	2.03		
IRAK1 rs1059703 G/G		224	220	2.139	0.343	221	212	4.472	0.107
A/G		48	57			69	85.82		
A/A		8	4			17	8		
TLR4 rs1927914 A/A		108	108	0.002	0.999	104	107	0.403	0.817
A/G		150	149			154	146		
G/G		51	51			46	50		
rs7873784 G/G		225	223	0.095	0.954	248	248	0.007	0.996
G/C		77	79			57	56		
C/C		8	7			3	3		

### 3.3 **Analysis of Gene Polymorphisms in RA Group and Control Group (**Table 3 **)**

**TABLE 3:** Logistic regression analysis of the SNPs of TNFAIP3, IRAK1, and TLR4 gene in RA and healthy controls.

SNPs	RA *n* = 308	Healthy controls *n* = 310	OR (95% CI)	*p* value
TNFAIP3 rs6920220	Allele A 5	5	0.993 (0.286–3.449)	0.992
	G 609	613		
Genotype	AA 2	2	1.003 (0.140–7.168)	0.997
	AG 1	3	0.334 (0.035–3.233)	0.344
	GG 304	305		
Recessive model	AA 2	2	0.990 (0.139–7.075)	0.992
Dominant model	AG + GG 305	308		
	AG + AA 3	5	1.661 (0.394–7.012)	0.485
	GG 304	305		
rs5029930				
Allele	C 60	30	0.469 (0.298–0.739)	0.001*
	A 554	590		
Genotype	CC 5	3	0.534 (0.126–2.258)	0.387
	AC 50	24	0.427 (0.255–0.716)	0.001*
	AA 252	283		
Recessive model	CC 5	3	0.590 (0.140–2.492)	0.468
Dominant model	AC + AA 302	307		
	AC + CC 55	27	0.437 (0.268–0.714)	0.001*
rs5029939	AA 252	283		
Allele	C 566	598	2.401 (1.436–4.017)	0.001*
	G 50	22		
Genotype	CC 264	290	3.295 (0.659–16.469)	0.124
	CG 38	18	1.421 (0.261–7.764)	0.683
	GG 6	2		
Recessive model	CC 264	290	2.417 (1.388–4.206)	0.001*
	CG + GG 44	20		
Dominant model	CG + CC 302	308	3.060 (0.613–15.278)	0.152
	GG 6	2		
IRAK1				
rs1059703				
Allele	A 103	64	0.640 (0.458–0.895)	0.009*
	G 511	496		
Genotype	AA 17	8	0.464 (0.196–1.098)	0.074
	AG 69	48	0.686 (0.454–1.037)	0.073
	GG 221	224		
Recessive model	AA 17	8	0.502 (0.213–1.181)	0.108
	AG + GG 290	272		
Dominant model	AA + AG 86	56	0.642 (0.437–0.944)	0.024*
	GG 221	224		
TLR4				
rs1927914				
Allele	A 362	366	0.987 (0.786–1.24)	0.910
	G 246	252		
Genotype	AA 104	108	0.937 (0.579–1.515)	0.790
	AG 154	150	0.87 (0.556–1.388)	0.579
	GG 46	51		
Recessive model	AA 104	108	1.033 (0.741–1.441)	0.847
	AG + GG 200	201		
Dominant model	AA + AG 258	258	0.902 (0.584–1.393)	0.641
	GG 46	51		
rs7873784				
Allele	C 63	93	1.549 (1.101–2.179)	0.012*
	G 553	527		
Genotype	CC 3	8	2.939 (0.77–11.215)	0.099
	CG 57	77	1.489 (1.011–2.193)	0.043*
	GG 248	225		
Recessive model	CC 3	8	2.693 (0.708–10.248)	0.131
	CG + GG 305	302		
Dominant model	CC + CG 60	85	1.561 (1.072–2.275)	0.020*
	GG 248	225		

Abbreviations: CI, confidence interval; n, number of members; OR, odds ratio; SNPs, single nucleotide polymorphisms.

* Represents *p* < 0.05, with statistical significance.

**FIGURE 1:**
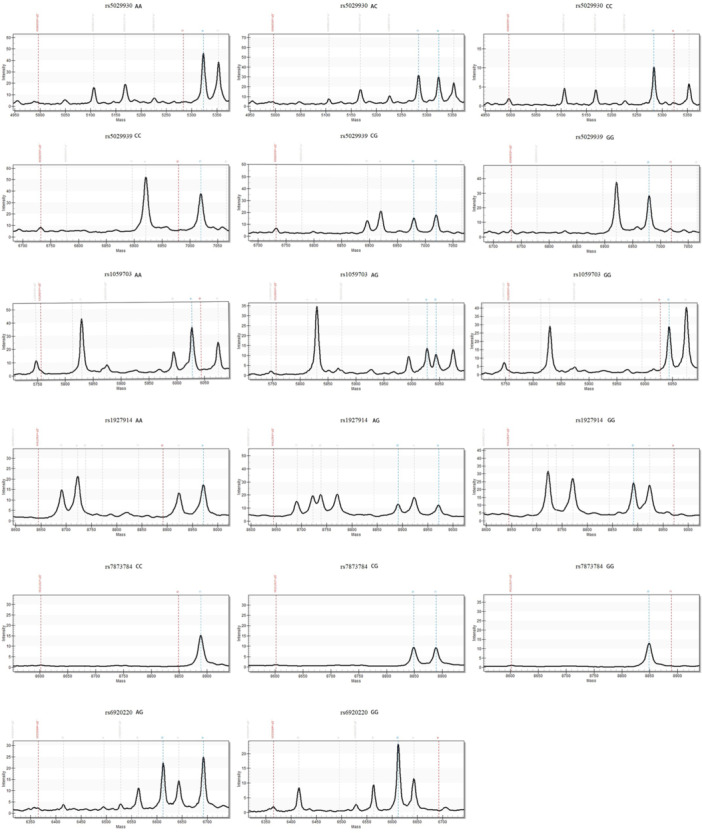
Mass spectrometry profiles for rs5029930, rs5029939, rs1059703, rs1927914, rs7873784, and rs6920220 loci.

MassARRAY genotyping mass spectra for each gene locus are shown in Figure 1.

In the TNFAIP3 gene, the rs5029930 C allele was protective against RA (OR = 0.469; 95% CI: 0.298–0.739; *p* = 0.001), whereas the rs5029939 C allele increased disease risk (OR = 2.401; 95% CI: 1.436–4.017; *p* = 0.001). Notably, rs5029930 AC genotype carriers showed reduced RA susceptibility compared to AA homozygotes (OR = 0.427; 95% CI: 0.255–0.716; *p* = 0.001), while rs5029939 CC genotypes were enriched in RA patients (OR = 2.417; 95% CI: 1.388–4.206; *p* = 0.001). A comparative analysis of the rs6920220 locus revealed no statistically significant variations in either allelic frequencies or genotypic profiles when comparing RA patients to healthy cohorts (*p* > 0.05).

In the IRAK1 gene, the A allele frequency at the rs1059703 locus decreased significantly in the RA group (OR = 0.640; 95% CI: 0.458–0.895; *p* = 0.009). The AA + AG phenotype frequency was significantly lower in the RA group compared to the healthy control group in relation to the GG genotype(OR = 0.642; 95% CI = 0.437–0.944; *p* = 0.024).

In the TLR4 gene, for rs1927914, the allele and genotype frequency were not statistically between the two groups (*p* > 0.05). The C allele frequency at the rs7873784 was significantly higher in the RA group (OR = 1.549; 95% CI: 1.101–2.179; *p* = 0.012). When using the GG genotype as a reference, the CG phenotype frequency was significantly increased in the group with RA (OR = 1.489; 95% CI: 1.011–2.193; *p* = 0.043). In the dominant model, which includes genotype GG, the CC + CG phenotype frequency was significantly higher in the group with RA (OR = 1.561; 95% CI = 1.072–2.275; *p* = 0.020).

### 3.4 Stratified Analysis

Supporting Information Tables 2 and 3 illustrate the differences in TNFAIP3, IRAK1, and TLR4 gene polymorphisms in RA considering sex and age. The rs5029930 (*p* = 0.006) in the TNFAIP3 gene and the rs1059703 (*p* = 0.000) in the IRAK1 gene exhibited significant differences in relation to sex across genotypes. The rs1927914 of the TLR4 gene exhibited notable variations in relation to age across different genotypes (*p* = 0.024). The differences among genotypes at the remaining loci were not statistically significant concerning age and sex (*p* > 0.05).

Supporting Information Tables 4 and 5 illustrate the correlation between TNFAIP3, IRAK1, and TLR4 gene polymorphisms and DAS28, GH, RF, and ACPA levels in RA. A notable disparity in DAS28 scores was found among the genotypes at the rs5029930 locus within the TNFAIP3 genes (*p* = 0.000), as well as a striking difference in RF‐positive titers among genotypes at the rs5029939 locus (*p* = 0.003). No striking differences in DAS28, GH, RF, and ACPA levels were found among genotypes at all other loci (*p* > 0.05).

The correlation of TNFAIP3, IRAK1, and TLR4 gene polymorphisms with the clinical manifestations of RA was further examined in Supporting Information Table 6. Polymorphisms at the rs7873784 locus of the TLR4 gene were correlated with the number of joint pressure pains across different genotypes (*p* = 0.048). Conversely, other genotypes were not significantly correlated with the number of joints exhibiting morning stiffness, pressure pain, or swelling (*p* > 0.05).

## 4 Discussion

Genetic factors significantly influence the development of RA, with an estimated heritability exceeding 50% (53%–60%) [22, 23, 24]. This research aims to examine the correlation between the polymorphisms of the TNFAIP3, IRAK1, and TLR4 genes and the susceptibility to RA, as well as its clinical characteristics in the Han Chinese population.

TNFAIP3, also referred to as A20, is situated on the q23 region of human chromosome 6 and functions as a suppressor of TNF‐κB activation triggered by TNF [25, 26]. Genetic variations in TNFAIP3 have been implicated in various autoimmune disorders, such as systemic sclerosis, systemic lupus erythematosus (SLE), and RA.

Currently, the TNFAIP3 gene locus rs5029930 and its association with RA have been reported only in a research conducted by Kim et al. [27]. The study revealed a lack of association between rs5029930 and RA susceptibility, and no correlation was found between the rs5029930 polymorphism and RA‐associated autoantibodies in the subgroup analysis. However, our research indicates that the rs5029930 polymorphism is linked to RA risk, and the allele C, genotype AC, and AC + CC models decreased the risk of RA. Different genotypes of rs5029930 were found to be associated with gender and DAS28 scores of RA in the stratified analysis. This discrepancy may stem from ethnic‐specific linkage disequilibrium patterns or gene‐environment interactions. These findings may serve as a reference for further research on the relationship between TNFAIP3 and RA, as well as for identifying new therapeutic targets in the future.

The rs5029939 locus of the TNFAIP3 gene has been extensively studied in relation to various immune disorders, particularly SLE. Previous research has demonstrated that the rs5029939 polymorphism is linked to genetic susceptibility to systemic sclerosis [28, 29], dermatomyositis or polymyositis [30], but not to desiccation syndrome [31]. Currently, only two studies have examined the association between the rs5029939 polymorphism and RA. One study by Kim found no association between the rs5029939 SNP and RA, but did find a strong association with SLE susceptibility and different joint phenotypes in SLE patients [27]. On the contrary, Hegab et al. observed no linkage between the rs5029939 polymorphism and RA, but did find associations with ACPA‐negative and positive phenotypes [32]. Our findings, however, are inconsistent with these previous studies. We found that there was an association between SNPs at the rs5029939 locus and RA susceptibility, with allele C and genotype CC increasing the risk of RA. Furthermore, different genotypes were associated with RF‐positive titers in RA patients. These varying findings may be associated with differences in ethnicity, environmental factors, and other variables.

For the rs6920220 locus of the TNFAIP3 gene, a study conducted in the Netherlands indicated that different genotypes of rs6920220 were not significantly linked to radiographic articular destruction in RA [33]. Ciccacci et al. demonstrated, based on an Italian study, that variant alleles of rs6920220 had a significant association with susceptibility to RA and SLE [34]. Another study involving 141 Tunisian patients with RA showed that the A allele at the rs6920220 loci had a risk effect for RA [35]. A study by Stark et al. conducted in the UK showed no correlation between rs6920220 and RA patients, which is consistent with our study [36]. In a Spanish study, it was found that rs6920220 was significantly linked to RA patients who tested positive for RF or ACPA [37]. A meta‐analysis conducted by Lee showed that the SNP of rs6920220 was only associated with European patients with RA [38]. Another meta‐analysis also demonstrated an association between the rs6920220 polymorphism and RA, identifying an increased risk of RA in Caucasian individuals [39]. In contrast to the results of our study, which found no correlation between the rs6920220 polymorphism and the risk of RA, subgroup analyses did not reveal a correlation between the rs6920220 polymorphism and clinical symptoms, DAS28 score, and GH. Considering that the inconsistency of specimens and experimental methods can affect the distribution of genetic polymorphisms, as well as the influence of racial heterogeneity and regional ethnicity on genetic polymorphisms, the relevant conclusions need to be further validated with larger sample sizes.

IRAK1 is situated at the Xq28 locus. This gene is activated by IL‐1, a powerful cytokine that is involved in inflammation and immune regulation [40]. IRAK1 encodes a serine/threonine kinase that functions as an indispensable component in the signal transduction cascades of TLRs and Interleukin‐1 receptors (IL1Rs). It serves as a critical mediator in the initial phases of the inflammatory response, helping to synthesize pro‐inflammatory cytokines, thus enhancing the immune response and coordinating the recruitment and activation of immune cells at the site of inflammation.

Recently, a study conducted on the Korean population demonstrated a strong correlation between rs1059703 and RA, both in allele and genotype models [41]. This finding underscores the potential role of the IRAK1‐mediated signaling axis as a fundamental pathophysiological process underpinning autoimmune disorders. Similarly, a study in Tunisia and France also reported a notable rise in the frequency of the rs1059703 major allele in RA patients compared to a control group, further reinforcing the link between IRAK1 polymorphisms and autoimmune conditions [42]. In addition, a study conducted in Iran revealed that the T allele of IRAK1 rs1059703 elevates both the risk and severity of RA [43]. Similarly, a Chinese meta‐analysis also demonstrated a significant correlation between rs1059703 and an elevated risk of RA, particularly among Caucasian populations [17]. However, previous studies have yielded inconsistent conclusions, with one study failing to find an association between IRAK1 and RA [44].

In our present study, a notable disparity was observed in the frequency of the rs1059703 allele A among the RA group and control groups, suggesting that rs1059703 is indeed associated with RA and that allele A may confer a protective effect, reducing the risk of RA. Several factors contribute to the discrepancies in the findings of these studies, including variations in sample sizes, statistical methodologies employed for sample size calculations, differences in populations based on region and ethnicity, and varying selection criteria for RA patients. Furthermore, the complex pathogenesis of RA, influenced by multiple SNPs, may involve interactions between SNPs themselves and between SNPs and environmental factors. Notably, the incidence of RA in women is approximately 2–3 times higher than in men, potentially due to the influence of estrogens. Considering that IRAK1 is a gene situated on the X chromosome, we analyzed the gender of patients with RA in relation to the genotype of the rs1059703 locus. The results indicated a significant association between gender and the genotype of the rs1059703 locus, further adding to the complexity of the genetic underpinnings of RA.

TLR has a key function in innate immune response. Both TLR and its ligands are implicated in disrupting tolerance to autoantigens and triggering a cascade of inflammatory reactions, which are crucial steps in the pathogenesis of RA [45, 46]. Enhanced TLR4 expression has been observed in diverse immune cells derived from individuals affected by distinct autoimmune disorders.

Specifically, the rs7873784 locus, situated within the 3′‐untranslated region of the TLR4, has been closely linked to the occurrence of numerous diseases, including RA. A study conducted on the Chinese population revealed that the TLR4 gene may represent a susceptibility gene for RA among the Han ethnicity in China [47]. The presence of the G/C polymorphism at TLR4 rs7873784 was found to decrease the risk of RA among patients. However, a genotyping study involving 213 RA patients, focusing on the SNP rs7873784 located in the 3′‐UTR, observed a significant genetic association between RA and rs7873784. It was hypothesized that the minor allele C of rs7873784 could elevate the risk of RA [48]. Paradoxically, another study exploring the relationship between RA and TLR4 rs7873784 in the Han population of central‐southern China found no correlation among the rs7873784 G/C polymorphism and RA risk [49]. However, our research shows that allele C and genotype CG may elevate the risk of RA. Stratified analysis revealed a statistically significant disparity in the quantity of compression joints among genotypes for the polymorphism of TLR4 rs7873784. The potential reasons for these findings could be as follows: First, regional, environmental, and dietary differences may lead to the inconsistency. Second, variations in sample sizes across studies may lead to conflicting conclusions. Third, clinical heterogeneity among patients could also play a role. Fourth, differences in the statistical methods employed in each case‐control study may contribute to varying results. Lastly, inconsistencies in the experimental methods used for genotyping could also be a factor.

The rs1927914 polymorphism of TLR4 is situated within the 5′‐ untranslated region of the TLR4 gene. This polymorphism may potentially influence the transcription factor binding site and regulate promoter activity, thereby modulating the inflammatory response and host immunity, and ultimately impacting susceptibility to RA [50]. The findings of this study did not identify a correlation between RA and rs1927914. Subgroup analysis showed that age could affect the TLR4 gene polymorphism of rs1927914.

There are certain limitations in this research. First of all, the relationship between all SNPs in the TNFAIP3, IRAK1, and TLR4 genes and RA was not explored. Additionally, the specific mechanisms through which these genes influence the clinical manifestations of RA were not thoroughly investigated. The absence of environmental exposure data (e.g., smoking and microbiome) restricts our capacity to evaluate gene‐environment interactions. Furthermore, the study did not explore the combined effects of multiple risk alleles, such as through genetic risk scoring or analysis of epistatic interactions among TNFAIP3, IRAK1, and TLR4 loci. Future studies with larger sample sizes are needed to investigate potential synergistic effects among these variants and to develop comprehensive genetic models for predicting RA risk.

In conclusion, our study suggests that TNFAIP3, IRAK1, and TLR4 gene polymorphisms are associated with RA susceptibility and might potentially help in future RA research.

## Author Contributions

**Zhenboyang Tang:** methodology, formal analysis, data curation, writing – original draft. **Lihui Peng:** methodology, formal analysis, data curation, writing – original draft. **Zixia Zhao:** formal analysis, investigation, data curation. **Xiping Zhou:** investigation, project administration. **Xiru Ling:** validation, data curation. **Chunyan Huang:** visualization, software. **Jiqiang Wu:** software, resources. **Ping Wang:** methodology, visualization. **Jie Chen:** supervision, writing – review and editing, conceptualization, funding acquisition.

## Ethics Statement

The study was agreed and supported by all the study subjects who signed the informed consent form. The study protocol was approved by the Ethics Research Committee of Southwest Medical University (KY2022152). All data and methods used in our study were in accordance with legal and ethical standards.

## Conflicts of Interest

The authors declare no conflicts of interest.

## Supporting information

Supplementary Information.docx.

## Data Availability

The data that support the findings of this study are available from the corresponding author upon reasonable request.
