# Supplementary material for: Analysis of TNFAIP3, IRAK1, and TLR4 Gene Polymorphisms in Patients With Rheumatoid Arthritis
Source: Immun Inflamm Dis. 2026 Feb 12;14(2):e70344. doi: 10.1002/iid3.70344 (PMC12902183; doi:10.1002/iid3.70344)
Supplement: Supplementary file 1 — Supplementary Information.docx. [file IID3-14-e70344-s001.docx]

**Analysis of TNFAIP3, IRAK1 and TLR4 gene polymorphisms in patients with rheumatoid arthritis**

**Supplementary Information**

supplement Table 1. Primer information of SNP in TNFAIP3, IRAK1and TLR4 gene

| SNP | enlarge the sequence of primers |
| --- | --- |
| rs6920220 | F: ACGTTGGATGGATCACTGTCTGCATATGCC |
|  | R: ACGTTGGATGTGCTTCCATCTGTTAGCAGG |
|  | S: ATTGTTCTACAGAACCATATC |
| rs5029930 | F: ACGTTGGATGTCCTGAGGAGTTTTCTGGTG |
|  | R: ACGTTGGATGAAGAGAGCACCATCTGATCC |
|  | S: GCTGGGAAAGGCATAG |
| rs5029939 | F: ACGTTGGATGTGACACCAACTGCAAAGGAG |
|  | R: ACGTTGGATGAATGCCCAGTGAACTTAAGG |
|  | S: TGGTACCTTGGTTCTAGCTTAA |
| rs1059703 | F: ACGTTGGATGACACGTAGGAGTTCTCCTGC |
|  | R: ACGTTGGATGTGTACGAGAGGCTAGAGAAG |
|  | S: TATTCAGCTGGCGGCCTCC |
| rs1927914 | F: ACGTTGGATGGGAAAGTAGCAAGTGCAATG |
|  | R: ACGTTGGATGGTGCTTGGAGGATATTACAG |
|  | S: GAACCTGATTTAAAACAGGAATATTATG |
| rs7873784 | F: ACGTTGGATGATGAGAGGTACCCTCTTAAC |
|  | R: ACGTTGGATGGCTCTAAAGATCAGCTGTAT |
|  | S: AGTGCATAATACAGTATTGTTCATTATA |

F: Upstream primers,R: Downstream primer,S: Extension primers.

supplement Table 2. Relationship between the genotype of TNFAIP3, IRAK1and TLR4 gene loci and sex

|  | | male | female | χ^2^ | *P* |
| --- | --- | --- | --- | --- | --- |
| TNFAIP3  rs6920220 | AA | 0 | 2 | 1.47 | 0.689 |
|  | AG | 0 | 1 |  |  |
|  | GG | 82 | 222 |  |  |
| rs5029930 | CC | 3 | 5 | 12.425 | 0.006* |
|  | AC | 24 | 50 |  |  |
|  | AA | 283 | 252 |  |  |
| rs5029939 | GG | 1 | 5 | 0.316 | 0.854 |
|  | CG | 10 | 28 |  |  |
| IRAK1 | CC | 71 | 193 |  |  |
| rs1059703 | AA | 11 | 6 | 24.8 | 0.000* |
|  | AG | 6 | 63 |  |  |
| TLR4 | GG | 65 | 156 |  |  |
| rs1927914 | AA | 29 | 75 | 1.525 | 0.466 |
|  | AG | 44 | 110 |  |  |
|  | GG | 9 | 37 |  |  |
| rs7873784 | CC | 0 | 3 | 2.32 | 0.313 |
|  | CG | 12 | 45 |  |  |
|  | GG | 70 | 178 |  |  |

* represents P < 0.05, with statistical significance.

supplement Table 3. Relationship between the genotype of TNFAIP3, IRAK1and TLR4 gene loci and age

|  | | mean | SD | *F* | *P* |
| --- | --- | --- | --- | --- | --- |
| TNFAIP3  rs6920220 | AA | 51.50 | 0.71 | 0.639 | 0.529 |
|  | AG | 65.00 | 13.15 |  |  |
|  | GG | 53.63 | 10.42 |  |  |
| rs5029930 | CC | 57.20 | 11.83 | 0.290 | 0.075 |
|  | AC | 53.70 | 10.94 |  |  |
|  | AA | 53.66 | 10.15 |  |  |
| rs5029939 | GG | 51.00 | 10.62 | 0.386 | 0.680 |
|  | CG | 54.66 | 11.19 |  |  |
| IRAK1 | CC | 53.55 | 10.28 |  |  |
| rs1059703 | AA | 57.29 | 10.95 | 1.115 | 0.329 |
|  | AG | 53.32 | 10.85 |  |  |
| TLR4 | GG | 53.47 | 10.20 |  |  |
| rs1927914 | AA | 55.68 | 9.35 | 3.766 | 0.024* |
|  | AG | 52.13 | 10.44 |  |  |
|  | GG | 54.15 | 11.72 |  |  |
| rs7873784 | CC | 54.00 | 4.58 | 0.519 | 0.596 |
|  | CG | 54.89 | 9.62 |  |  |
|  | GG | 53.34 | 10.59 |  |  |

SD:Standard deviation; * represents P < 0.05, with statistical significance.

supplement Table 4. Relationship between the genotype of TNFAIP3, IRAK1and TLR4 gene loci and DAS28 and GH

|  | | DAS28 | | | | GH | | |
| --- | --- | --- | --- | --- | --- | --- | --- | --- |
|  |  | 1 | 2 | 3 | *P* | mean | SD | *P* |
| TNFAIP3  rs6920220 | AA | 0 | 1 | 1 | 0.894 | 60.00 | 16.14 | 0.326 |
|  | AG | 0 | 0 | 1 |  | 80.00 | 14.29 |  |
|  | GG | 6 | 73 | 225 |  | 74.56 | 14.19 |  |
| rs5029930 | CC | 2 | 1 | 2 | 0.000* | 64.00 | 18.17 | 0.245 |
|  | AC | 0 | 15 | 35 |  | 75.00 | 15.89 |  |
|  | AA | 4 | 58 | 190 |  | 74.58 | 13.71 |  |
| rs5029939  IRAK1 | GG | 1 | 1 | 4 | 0.067 | 70.00 | 20.25 | 0.681 |
|  | CG | 0 | 12 | 26 |  | 73.79 | 14.86 |  |
|  | CC | 5 | 61 | 198 |  | 74.74 | 13.97 |  |
| rs1059703  TLR4 | AA | 0 | 4 | 13 | 0.854 | 73.82 | 17.37 | 0.839 |
|  | AG | 2 | 19 | 48 |  | 73.68 | 14.35 |  |
|  | GG | 4 | 51 | 166 |  | 74.78 | 13.91 |  |
| rs1927914 | AA | 4 | 23 | 77 | 0.109 | 75.73 | 14.33 | 0.390 |
|  | AG | 1 | 43 | 110 |  | 75.35 | 14.31 |  |
|  | GG | 1 | 6 | 39 |  | 75.17 | 13.44 |  |
| rs7873784 | CC | 0 | 0 | 3 | 0.897 | 83.33 | 5.77 | 0.541 |
|  | CG | 1 | 14 | 42 |  | 74.88 | 12.92 |  |
|  | GG | 5 | 60 | 183 |  | 74.34 | 14.51 |  |

1 represents DAS28 score less than or equal to 3.2 , 2 represents DAS28 score greater than 3.2 less than or equal to 5.1,3 represents DAS28 score greater than 5.1;* represents P < 0.05, with statistical significance.

supplement Table 5. Relationship between Genotype of TNFAIP3, IRAK1and TLR4 Gene Loci and RF, ACPA

|  | | RF | | | ACPA | | |
| --- | --- | --- | --- | --- | --- | --- | --- |
|  |  | mean | SD | *P* | positive | negative | *P* |
| TNFAIP3  rs6920220 | AA | 77.15 | 26.23 | 0.399 | 2 | 0 | 0.874 |
|  | AG | 22.10 | 20.18 |  | 1 | 0 |  |
|  | GG | 214.98 | 201.37 |  | 279 | 25 |  |
| rs5029930 | CC | 255.30 | 127.09 | 0.891 | 4 | 1 | 0.533 |
|  | AC | 214.86 | 167.26 |  | 47 | 3 |  |
|  | AA | 212.00 | 208.79 |  | 231 | 21 |  |
| rs5029939  IRAK1 | GG | 486.97 | 603.77 | 0.003* | 6 | 0 | 0.760 |
|  | CG | 198.21 | 147.40 |  | 35 | 3 |  |
|  | CC | 208.74 | 188.10 |  | 242 | 22 |  |
| rs1059703  TLR4 | AA | 216.65 | 174.89 | 0.550 | 16 | 1 | 0.761 |
|  | AG | 236.36 | 258.41 |  | 62 | 7 |  |
|  | GG | 206.06 | 181.95 |  | 204 | 17 |  |
| rs1927914 | AA | 192.62 | 162.82 | 0.204 | 93 | 11 | 0.223 |
|  | AG | 233.72 | 230.31 |  | 141 | 13 |  |
|  | GG | 192.19 | 171.34 |  | 45 | 1 |  |
| rs7873784 | CC | 428.00 | 138.56 | 0.101 | 3 | 0 | 0.678 |
|  | CG | 236.11 | 269.80 |  | 51 | 6 |  |
|  | GG | 204.92 | 181.04 |  | 229 | 19 |  |

* represents P < 0.05, with statistical significance.

supplement Table 6. Relationship between Genotype of TNFAIP3, IRAK1and TLR4 Gene Loci and Tenderness, Swelling of Joints, and Morning Stiffness

|  | | Tender Joint | | | Swelling Joints | | | Morning Stiffness | | |
| --- | --- | --- | --- | --- | --- | --- | --- | --- | --- | --- |
|  |  | mean | SD | *P* | mean | SD | *P* | no | yes | *P* |
| TNFAIP3  rs6920220 | AA | 2.50 | 2.12 | 0.282 | 2.50 | 2.12 | 0.304 | 1 | 1 | 0.636 |
|  | AG | 16.00 | 11.21 |  | 16.00 | 11.21 |  | 0 | 1 |  |
|  | GG | 10.31 | 7.79 |  | 6.92 | 7.14 |  | 82 | 222 |  |
| rs5029930 | CC | 6.40 | 9.74 | 0.511 | 6.40 | 9.74 | 0.916 | 3 | 2 | 0.182 |
|  | AC | 10.66 | 8.39 |  | 6.62 | 7.14 |  | 11 | 39 |  |
|  | AA | 10.33 | 7.69 |  | 7.04 | 7.17 |  | 69 | 183 |  |
| rs5029939  IRAK1 | GG | 10.83 | 9.02 | 0.973 | 9.50 | 10.69 | 0.610 | 2 | 4 | 0.431 |
|  | CG | 10.11 | 7.94 |  | 6.39 | 6.59 |  | 7 | 31 |  |
|  | CC | 10.34 | 7.80 |  | 6.99 | 7.17 |  | 74 | 190 |  |
| rs1059703  TLR4 | AA | 10.47 | 8.80 | 0.994 | 6.71 | 6.20 | 0.539 | 9 | 8 | 0.052 |
|  | AG | 10.25 | 7.52 |  | 6.10 | 6.73 |  | 56 | 13 |  |
|  | GG | 10.27 | 7.83 |  | 7.19 | 7.32 |  | 159 | 62 |  |
| rs1927914 | AA | 10.79 | 7.70 | 0.350 | 7.47 | 7.18 | 0.246 | 77 | 27 | 0.278 |
|  | AG | 9.64 | 7.83 |  | 6.32 | 7.07 |  | 115 | 39 |  |
|  | GG | 11.17 | 7.74 |  | 8.04 | 7.21 |  | 29 | 17 |  |
| rs7873784 | CC | 19.00 | 4.58 | 0.048* | 11.33 | 10.26 | 0.376 | 2 | 1 | 0.655 |
|  | CG | 11.65 | 8.68 |  | 6.14 | 6.87 |  | 39 | 18 |  |
|  | GG | 9.91 | 7.56 |  | 7.10 | 7.21 |  | 184 | 64 |  |
